# Efficacy and safety of phototherapy for jaundice in full-term newborns: a meta-analysis

**DOI:** 10.3389/fped.2025.1701717

**Published:** 2026-06-24

**Authors:** Xingzhen Feng, Zijun Liang, Huishi Ye, Shubin Xie

**Affiliations:** Department of Paediatrics, Dongguan Hospital of Traditional Chinese Medicine, Dongguan, Guangdong, China

**Keywords:** phototherapy, jaundice, full-term neonates, therapeutic effect, meta-analysis

## Abstract

**Objective:**

The efficacy and safety of phototherapy in the treatment of full-term neonatal jaundice were meta-analyzed, and the advantages of phototherapy were summarized.

**Methods:**

PubMed, Embase, Cochrane Library, and Web of Science databases were searched using subject + free words for the subject words “phototherapy,” “jaundice,” and “full-term newborn,” and the search time was limited from 1 January 2000 to 25 April 2024, and the search language was unlimited. According to the inclusion criteria and exclusion criteria, two researchers independently conducted a three-level screening of the retrieved literature to obtain the included literature. Collected and compared information included in the literature. Review Manager 5.3 was used to plot bias plots, forest plots, and funnel plots. Statistical analyses were performed using IBM SPSS Statistics 26 and Stata 17 software.

**Results:**

We obtained a total of 486 literature entries, of which 17 were included in the systematic review. The meta-analysis included 647 full-term neonates from 14 studies with complete serum total bilirubin (STB) data before and after phototherapy. The pooled mean reduction in STB was 5.43 mg/dL (95% CI: 5.30–5.56). *χ*^2^ = 728.01, df = 13 (*p* < 0.00001), *I*^2^ = 98%, and *Z* = 80.54 (*p* < 0.00001) for STB (mg/dL) before and after phototherapy. The STB reduction rate in all studies was >18%.

**Conclusion:**

Proper phototherapy can significantly reduce the level of STB and eliminate the symptoms of jaundice in full-term neonates. Light-emitting diode phototherapy devices demonstrated higher efficacy compared with fluorescent tube systems. The treatment is safe with minimal adverse effects.

**Systematic Review Registration:**

PROSPERO.

## Introduction

According to the World Health Organization, a full-term newborn is defined as a live birth between 37 and 42 completed weeks of gestation (1, 2). Neonatal jaundice refers to the neonatal period (from umbilical cord ligation to 28 days after birth) in which bilirubin accumulates in the body, resulting in increased bilirubin levels in the blood. Symptoms include yellow discoloration of the skin, mucous membrane, sclera, and other organs (3, 4). The disease is divided into physiologic jaundice and pathological jaundice (5). Physiologic jaundice appears 2–3 days after birth and peaks at 4–5 days, and serum bilirubin generally does not exceed 220.6 μmol/L (12.9 mg/dL). It subsides by 5–7 days, and usually no later than 2 weeks. In preterm infants, it lasts longer, and serum bilirubin generally does not exceed 255 μmol/L (15 mg/dL) (6, 7). In addition to slight loss of appetite, there are generally no other clinical symptoms. If jaundice occurs 24 h after birth, fails to resolve within 2–3 weeks, shows a daily serum bilirubin increase of >85 μmol/L, continues to deepen and aggravate, recurs after fading, or begins to appear after 2 weeks after birth, it is considered pathological jaundice (8, 9). Pathological jaundice is characterized by hyperbilirubinemia, which means that the serum total bilirubin exceeds 220.6 μmol/L (12.9 mg/dL) and the peak exceeds 342 μmol/L (20 mg/dL) in full-term infants and neonates (10, 11).

When the indirect bilirubin exceeds 307.8 μmol/L (18 mg/dL) in full-term neonates, the risk of bilirubin-induced neurologic dysfunction (BIND) is significantly higher. Severe unconjugated hyperbilirubinemia leads to BIND; the chronic form, kernicterus, results from the deposition of bilirubin in the brainstem and basal ganglia nuclei. This condition typically occurs when indirect bilirubin levels exceed the brain's protective capacity, allowing bilirubin to cross the blood–brain barrier and cause irreversible damage to neurons. The clinical manifestations include acute bilirubin encephalopathy in the neonatal period and chronic bilirubin encephalopathy with long-term sequelae such as athetoid cerebral palsy, auditory neuropathy, gaze abnormalities, and dental enamel dysplasia. Unlike other forms of jaundice that primarily affect the skin and mucous membranes, bilirubin encephalopathy (kernicterus) represents a medical emergency requiring immediate intervention to prevent permanent neurological damage. The primary causes include severe hemolytic disease, prematurity with immature liver function, and genetic disorders affecting bilirubin metabolism, such as Crigler–Najjar syndrome (12, 13).

Physiologic jaundice usually does not need treatment; strengthening feeding and increased bowel movements can help resolve it naturally (14). Pathological jaundice requires phototherapy, massage, drug treatment, and other interventions, but it is essential to identify and treat the primary cause of jaundice (15, 16). At present, the main treatment method for neonatal unbound (indirect) bilirubin elevation is continuous blue light irradiation therapy to promote unbound bilirubin excretion, while real-time monitoring of serum bilirubin levels during the course of phototherapy (normal range for full-term neonates: total bilirubin <12.9 mg/dL, indirect bilirubin <11.3 mg/dL, direct bilirubin <1.5 mg/dL) (17, 18). The use of phototherapy for neonatal hyperbilirubinemia was first proposed in 1958 by Dr. Cremer, head nurse at Rochford General Hospital in Essex, UK, and his colleagues. The principle of phototherapy is to irradiate the skin with specific wavelengths of light (blue light with wavelength 425–475 nm and green light with wavelength 510–530 nm) and convert unbound bilirubin into water-soluble isomers under the action of light, which can be discharged from the body through bile and urine, thereby reducing serum unbound bilirubin and reducing or eliminating jaundice symptoms (19, 20). According to the American Academy of Pediatrics (AAP) Clinical Practice Guideline (21), phototherapy is recommended when total serum bilirubin levels approach or exceed age-specific thresholds based on risk factors and postnatal age in hours. The adverse reactions of phototherapy include fever, hypothermia or diarrhea, rash, and even DNA damage, although these typically disappear after stopping phototherapy (22, 23). In the process of phototherapy, attention should be paid to supplementing fluid, ensuring adequate urine discharge, and monitoring the temperature of the child. In addition to phototherapy, intravenous immunoglobulin is also used in clinical treatment, along with drugs such as albumin, ursodeoxycholic acid, phenobarbital, and clofibrate.

In recent years, several studies have discussed the safety of phototherapy in the treatment of neonatal jaundice. Bulut and Duruyen (24) demonstrated that strong light-emitting diode (LED) phototherapy has no effect on the DNA of full-term neonates (2–8 days of birth), nor does it cause oxidative stress. The oxidative marker of DNA damage in plasma, 8-hydroxy-2-deoxyguanosine (8-OH-dG), had no significant changes compared with that before phototherapy (*P* > 0.05). van der Schoor et al. (25) found that the use of blue LED phototherapy (BLP) at 35 µW/cm^2^/nm in preterm infants ≤32 weeks gestation did not affect 8-OH-dG levels. These results all support the safety of phototherapy in the treatment of full-term neonatal jaundice. In this study, we conducted a meta-analysis of the efficacy and safety of phototherapy for neonatal pathological jaundice, comparing different phototherapy devices and treatment protocols to provide comprehensive clinical guidance for the treatment of neonatal jaundice.

## Methods

### Literature search and screening

This systematic review and meta-analysis was conducted in accordance with the 2020 Preferred Reporting Items for Systematic Reviews and Meta-Analyses (PRISMA) guidelines. The study was registered with PROSPERO. Four databases, namely, PubMed, Embase, Cochrane Library, and Web of Science, were searched for topic words and free words (26). Similar systematic review and meta-analysis methodologies have been successfully applied in various clinical domains (27–29). The deadline is 22 April 2024, with no language restrictions. The MeSH Terms are phototherapy, full-term neonates, and jaundice. The search terms are as follows: ((“phototherapy” [MeSH Terms] OR “Low-Level Light Therapy” [All Fields] OR “Ultraviolet Therapy” [All Fields]) AND (“newborn” [MeSH Terms] OR “infant, newborn” [All Fields] OR “full term” [All Fields] OR “newborn infant” [All Fields]) AND (“jaundice” [MeSH Terms] OR “jaundiced” [All Fields] OR “jaundices” [All Fields])). Two researchers independently conducted a three-level screening of the retrieved literature according to the inclusion and exclusion criteria, removing duplicate literature, deleting literature through preliminary reading, and excluding literature after thorough reading. The inclusion criteria are as follows: (1) literature related to phototherapy for neonatal jaundice; (2) full-term neonates; and (3) research focusing solely on phototherapy. The exclusion criteria are as follows: (1) duplicate literature; (2) unable to obtain full text; (3) incomplete data; (4) overview, guidelines, meta-analysis, or research on risk factors; (5) only involving massage, exchange transfusion, and medication treatment; (6) the operating methods and techniques of phototherapy; (7) non-term, premature infants; (8) bilirubin evaluation methods, percutaneous bilirubin measurement method, and bilirubin prediction for jaundice; (9) not related to jaundice, such as phototherapy affecting kidney function, oxygen consumption, antioxidant stress, encephalopathy, neuropathy; (10) ABO hemolysis; (11) not related to phototherapy, such as only discussing the effects of headsets and eye masks, the effects of breast milk, and skincare; (12) only discussing the side effects of phototherapy, such as dermatitis, skin dehydration, and skin sensitivity; (13) pregnancy-related and delivery-related; (14) non-neonatal, only exploring indicators of childhood or adulthood. The main outcome of this study was the comparison of neonatal STB (mg/dL) before and after phototherapy. Secondary outcomes were bias analysis and heterogeneity in the included literature. We applied the Grading of Recommendation, Evaluation, Development and Evaluation (GRADE) approach to assess the certainty of the evidence in accordance with the guidelines of the Cochrane Handbook.

### Extract information included in the literature

We extracted and compared the information in the included literature, analyzing only measurements of unilateral or unidirectional continuous phototherapy. We used the Population, Intervention, Comparison, Outcome, and Study Design (PICOS) framework to perform a tabular analysis of the population, interventions, comparisons, outcomes, and study design. The following data were extracted: author and year of publication, number of neonates with phototherapy, gestational age (weeks), days after birth, birth weight (g), weight before phototherapy (g), gender distribution, cesarean delivery rate, phototherapy device, phototherapy time, STB before phototherapy (mg/dL), STB after phototherapy (mg/dL), and study groups. The efficacy and safety of phototherapy in the treatment of term neonatal jaundice were compared and summarized across all the literature.

### Bias analysis

Review Manager 5.3 software was used to assess the bias of the included literature. By selecting “yes,” “no,” or “unclear” for various questions in the four domains of patient selection, index tests, reference standard, and flow and timing, the risk of bias of the included literature was analyzed. Each included paper was read closely, and each question was answered systematically.

### Forest plots and comparison of indicators

Forest plots were drawn using Review Manager 5.3 to display the effect size and confidence interval (CI) of each included study. The plots describe the mean difference and confidence intervals using multiple line segments parallel to the horizontal axis, centered on a vertical line representing no effect. When the studied event is a “favorable event,” the system default settings were modified so that the left side of the horizontal coordinate represents “favors control” and the right side represents “favors treatment”.

### Funnel plot

Funnel plots were drawn with Review Manager 5.3 to assess publication bias. The effect size (mean difference) was used as the horizontal coordinate, and the standard error (SE) was used as the vertical coordinate. The symmetry of the funnel plot indicates the presence or absence of publication bias.

### Sensitivity analysis

Given the high heterogeneity (*I*^2^ > 75%) in the forest plot, sensitivity analysis was conducted using Stata 17 software with the one-by-one elimination method. Each included reference was excluded in turn, and the remaining references (*n* − 1) were combined for meta-analysis to assess whether there were significant changes in the combined results.

### Meta-regression analyses

Meta-regression analysis using Stata 17 software was performed to evaluate the size and source of heterogeneity. This analysis explored the influence of covariables such as year of publication, total number of patients, gestational age, birth weight, gender, cesarean delivery, and phototherapy time on the combined effect.

### Statistics

IBM SPSS Statistics 26 software was used to calculate the measured values of phototherapy effects. Dichotomous variables were represented by *n* (%), and the chi-square test was used to detect statistical differences between multiple items. Continuous variables were represented by *x* ± s (mean ± standard deviation), and statistical differences between two items were detected by a *t*-test. *P* < 0.05 was considered statistically significant. Review Manager 5.3 software was used to plot statistical results. Stata 17 software and Comprehensive Meta-Analysis (CMA) software were used to perform sensitivity analysis and meta-regression analyses.

## Results

### Results of the literature search and screening

**Figure 1:**
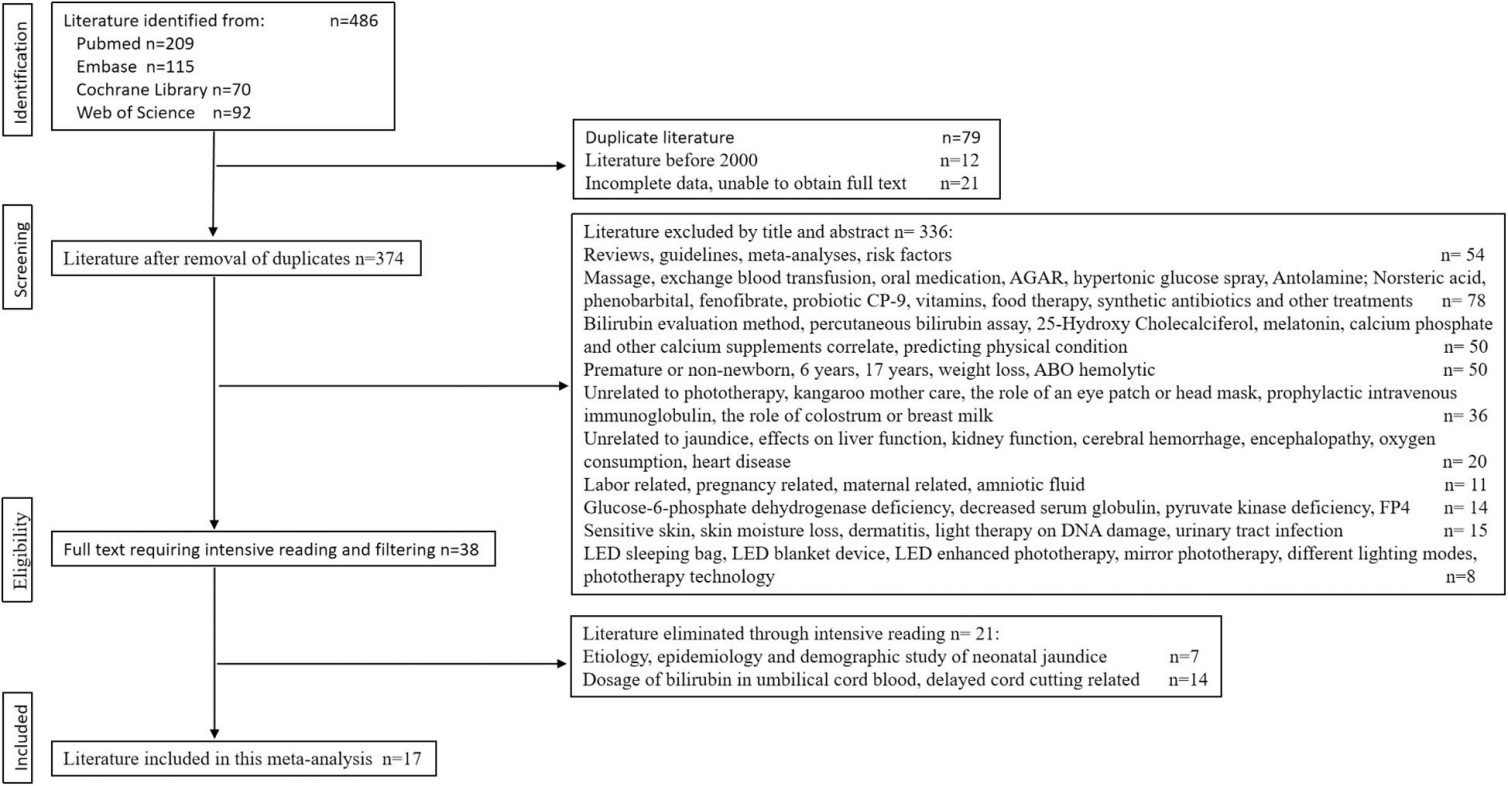
Literature screening process. A total of 486 literature entries were retrieved. After conditional screening, preliminary reading screening, and intensive reading screening, 17 studies were included in the systematic review. Among these, 14 studies had complete STB data before and after phototherapy and were included in the meta-analysis, while 3 studies lacked complete bilirubin data and were included only in the systematic review.

Through the search, we obtained a total of 486 literature entries: 209 from PubMed, 115 from Embase, 70 from Cochrane Library, and 92 from Web of Science. After the first round of basic screening, 79 duplicate entries were removed, 12 were published before 2000, and 21 were excluded due to unavailable with full text or incomplete data. A second preliminary reading was performed on the remaining 374 literature entries, excluding 54 reviews, guidelines, meta-analyses, and risk factor studies. Additionally, 78 entries discussing treatment methods other than phototherapy (such as massage, exchange transfusion, and oral drugs), 50 discussing bilirubin evaluation methods and physical condition prediction, 50 related to premature infants or non-neonates and ABO hemolysis, 36 unrelated to phototherapy, and 20 unrelated to jaundice were excluded. There were 11 articles related to childbirth and maternal factors, 14 related to causes such as glucose-6-phosphate dehydrogenase deficiency, 8 related to LED sleeping bag and other phototherapy methods and technologies, and 15 related to skin sensitivity, skin moisture loss, dermatitis, DNA damage, and other adverse reactions of phototherapy. After intensive reading of the remaining 38 literature entries, 7 focusing on etiology, epidemiology, and demography of neonatal jaundice and 14 related to cord blood bilirubin and delayed cord cutting were removed. Finally, 17 literature entries were included in the systematic review, of which 14 studies had complete STB (mg/dL) data before and after phototherapy, as shown in Figure 1. The included literature comprises the following: Silva et al. (30), Sachdeva et al. (31), Yahia et al. (32), Mosayebi et al. (33), Das et al. (47), El-Sheikh et al. (34), Nazim et al. (48), Aycicek and Erel (49), Kale et al. (50), Eyada et al. (51), Eldondity et al. (52), Iskander et al. (35), Boonyarittipong et al. (36), Shimada et al. (37), Kurban et al. (53), Mirnia et al. (54).

### Comparison of information included in the literature

The following data were extracted from the included literature: author and year of publication, number of neonates with phototherapy, gestational age (weeks), days after birth, birth weight (g), weight before phototherapy (g), gender distribution (female *n*/%, male *n*/%), cesarean delivery (*n*/%), phototherapy device, phototherapy time, STB before phototherapy (mg/dL), STB after phototherapy (mg/dL), and study group descriptions. Table 1 shows the comparison of the specific characteristics of each included study. The values of STB (mg/dL), an important marker of jaundice in full-term neonates, were compared before and after phototherapy, as shown in Table 2.

**Table 1:** Basic information in the included literature and research.

Author and year of publication	Number of newborns with phototherapy^a^	Gestational age (weeks)	Days after birth	Birth weight (g)	Weight before phototherapy (g)	Gender/female (*n*/%)	Gender/male (*n*/%)	Cesarean delivery (*n*/%)	Phototherapy device	Phototherapy time	Serum total bilirubin before phototherapy (mg/dL)	Serum total bilirubin after phototherapy (mg/dL)	Study group^b^
Silva et al. (2009) (30)	37	38.4 ± 0.8	–	3,424 ± 511	3,211 ± 448	17/46	20/54	–	Six to eight fluorescent tubes (two blue and four white 20 W lamps in each tube). positioned at 30-40 cm from the patients	24h	16.7 ± 1.7	13.5 ± 0.36	Single phototherapy vs. double phototherapy
Sachdeva et al. (2015) (31)	39	37 ± 1.1	4.13 ± 1.58	2,908 ± 455	2,901.9 ± 122.14	19/48.7	20/51.3	11 (28.2)	Single overhead compact fluorescent phototherapy unit (Phoenix Medical Systems Pvt. Ltd.). A distance of 25–30 cm was maintained between the baby and the bulb/lamp surface. A minimum of 30 μW/cm^2^/nm	30 (24–42)h	19.3 ± 1.5	15.4 ± 1.6	Intermittent (IPT) vs. continuous (CPT) group
Yahia et al. (2015) (32)	45	37.9 ± 1.1	4.8 ± 1.9	2.9 ± 1.2	±	26/57.8	19/47.5	19/47.5	Phototherapy system (phototherapy unit; 520 Cobams srl, Bologna, Italy) consisted of six fluorescent special blue light lamps (Philips F20T12/BB). The phototherapy intensity (=10 μW/cm^2^/nm) and spectrum were centered approximately 450–560 nm, at a distance of 40 cm	12 h	17.4 ± 1.1	10.1 ± 1.5	Infants with hyperbilirubinemia requiring phototherapy vs. infants with physiological jaundice not requiring phototherapy vs. infants without clinical jaundice. vs
Mosayebi et al. (2016) (33)	128	38.5 ± 0.6	5.7 ± 2.9	3,163 ± 362	3,002 ± 370	/	64/50	81/63	–	57.6 ± 14.4 h	18.5 ± 2.9	–	Full-term exclusively breastfed newborns with non-hemolytic jaundice
Das et al. (2024) (47)	35	37.63 ± 0.84	3.89 ± 1.13	2,410 ± 280	–	–	–	–	The Seefar Nice 4000 Spot Light-emitting Diode (LED) phototherapy machine, a microprocessor-controlled system with 24 Hi Bright Blue LED lamps and 3 white LED lamps. The irradiance level was maintained at 30 μW/cm^2^/nm at a distance as close as possible	96 h	14.69 ± 1.53	13.16 ± 1.49	Receiving phototherapy and vitamin D vs. receiving phototherapy alone
El-Sheikh et al. (2022) (34)	100	38.44 ± 1.03	3.36 ± 1.6	–	–	40/40	60/60	80/80	–	72 h	17.21 ± 1.74	8.85 ± 0.62	Full-term infants with indirect hyperbilirubinemia in the first 2 weeks
Nazim et al. (2023) (48)	100	39 ± 1	5 ± 1	–	3,000 ± 500	48/48	52/52	30/30	The Phoenix LED phototherapy units used for the treatment emitted light in the blue-green spectrum	48 h	17.5 ± 2.1	12.5 ± 1.8	100 term neonates
Mosayebi et al. (2016) (33)	143	38.52 ± 0.690	5.89 ± 3.118	3,166.68 ± 363.146	3,018.04 ± 375.788	71/49.7	72/50.3	89/62.2	–	59.28 ± 16.32h	18.46 ± 2.84	–	Mild bilirubin(<18 mg/dL) group vs. moderate bilirubin(18–20 mg/dL) group vs. high bilirubin(>20 mg/dL) group
Aycicek and Erel (2007) (49)	57	–	6 ± 3	–	3,100 ± 1,400	28/49.12	29/50.88	–	A phototherapy system consisting of six white fluorescent tubes (Philips TL 20 W/54) 40 cm above	48 h	17.1 ± 2.5	13.8 ± 2.3	Full-term infants from 3–10 days of age exposed to phototherapy
Kale et al. (2013) (50)	29	38 (35–40)	5.67 ± 1.2	3,060 ± 464	–	11/37.93	18/62.07	13/45	The IC-100 Phototherapy System (Ertunç Özcan, Ankara) consisting of four blue ﬂuorescent lamps (Osram L 18 W/67 Lumilux Blue, Germany, intensity: 10–15 μW/cm^2^/nm, spectrum 430–470 nm)	24 h	19 ± 2.4	12.8 ± 2.4	Conventional phototherapy group vs. LED phototherapy group vs. fiber-optic phototherapy group
Eyada et al. (2017) (51)	25	38 (37–40)	≤14	–	3,200 (2,500- 3,900)	10/40	15/60	13/52	White ﬂuorescent lamps emitting light at a wavelength of 420–470 nm and placed at 40 cm from the neonates	72 h	19.5 (14.8–24.2)	8.9 (6.5–11)	Indirect hyperbilirubinaemia and treated with conventional phototherapy vs. healthy matched neonates
Eldondity et al. (2021) (52)	30	37 ± 0.45	3 ± 1.35	–	–	14/46.67	16/53.33	25/83.3	–	48 h	14.0 (12.8–16.2)	9.15 (6.8–11.7)	30 full-term neonates with indirect hyperbilirubinemia requiring phototherapy
Iskander et al. (2021) (35)	40	38.2 ± 0.4	3.93 ± 1.54	±	3,104 ± 420	17/42	23/58	18/45	The device consists of a chamber containing 16 blue TL 20 W/52 fluorescent tubes arranged cylindrically. The distance between the infant and the lights is 30 cm from all sides	8 h	23.4 ± 4.2	15.4 ± 3.4	Term newborns with severe jaundice vs. non-jaundiced apparently normal controls
Boonyarittipong et al. (2008) (36)	30	38.7 + 1.3	–	3,105.0 + 293.6	2,928.3 + 283.4	12/40	18/60	–	The SsIPT consisting of four deep blue (Toshiba Lighting FL18W/T8/DB) and two daylight (Toshiba Lighting FL18W/T8/D) fluorescent lamps, placed at least 30 cm above the infant	48 h	14.8 + 1.7	8.4 + 1.7	Group 1 infants received single-surface intensive phototherapy vs. Group 2 infants received double-surface intensive phototherapy
Shimada et al. (2003) (37)	10	38.9 ± 1.0	1.84 ± 0.08	2,472 ± 420	–	–	–	–	High-intensity light, ranging from 3,000 (bank phototherapy lights) up to 20,000 lux (halogen spot lights)	31.5 ± 16.4 h	–	–	Infants treated with phototherapy vs. untreated infants
Kurban et al. (2014) (53)	40	38.42 ± 1.34	5.90 ± 2.52	–	3,000 ± 400	21/52.5	19/47.5	–	16 white fluorescent tubes (Philips TL 20W/52) and 40 cm above. The light energy of the phototherapy unit, measured by a standard photometer, was 22–26 µW/cm^2^/nm	29.7 ± 8.6h	21.84 ± 3.6	11.29 ± 1.3	Full-term newborns exposed to phototherapy
Mirnia et al. (2023) (54)	40	>38	6 ± 3	3,332.13 ± 367.359	–	21/52.5	19/47.5	–	The phototherapy device was equipped with Tosan-manufactured LED lamps, from Iran, that emitted light with a wavelength of 400–500 nm. The distance between the newborn and the lamp was 50 and 15–20 cm	72 h	22.12 ± 2.47	12.52 ± 2.63	40 full-term newborns with hyperbilirubinemia

^a^ The data in this table are for groups of simple, unidirectional, or unilateral continuous phototherapy included in the literature.

^b^ This list provides an overview of the study groups included in the literature.

**Table 2:** Comparison of various detection values before and after phototherapy in the included studies.

Author and year of publication	Number of full-term newborns with single-direction continuous light therapy	Evaluation indicators	Before the phototherapy	After the phototherapy	Serum total bilirubin decline rate (%)
Silva et al. (2009) (30)	37	Serum total bilirubin (mg/dL)	16.7 ± 1.7	13.5 ± 0.36	−19.16
Sachdeva et al. (2015) (31)	39	Serum total bilirubin (mg/dL)	19.3 ± 1.5	15.4 ± 1.6	−20.21
Yahia et al. (2015) (32)	45	Serum total bilirubin (mg/dL)	17.4 ± 1.1	10.1 ± 1.5	−41.95
Mosayebi et al. (2016) (33)	128	Serum total bilirubin (mg/dL)	18.5 ± 2.9	–	–
Das et al. (2024) (47)	35	Serum total bilirubin (mg/dL)	14.69 ± 1.53	13.16 ± 1.49	−10.42
El-Sheikh et al. (2022) (34)	100	Serum total bilirubin (mg/dL)	17.21 ± 1.74	8.85 ± 0.62	−48.58
Nazim et al. (2023) (48)	100	Serum total bilirubin (mg/dL)	17.5 ± 2.1	12.5 ± 1.8	−28.57
Mosayebi et al. (2016) (33)	143	Serum total bilirubin (mg/dL)	18.46 ± 2.84	–	–
Aycicek and Erel (2007) (49)	57	Serum total bilirubin (mg/dL)	17.1 ± 2.5	13.8 ± 2.3	−19.30
Kale et al. (2013) (50)	29	Serum total bilirubin (mg/dL)	19 ± 2.4	12.8 ± 2.4	−32.63
Eyada et al. (2017) (51)	25	Serum total bilirubin (mg/dL)	19.5 (14.8–24.2)	8.9 (6.5–11)	−54.36
Eldondity et al. (2021) (52)	30	Serum total bilirubin (mg/dL)	14.0 (12.8–16.2)	9.15 (6.8–11.7)	−34.64
Iskander et al. (2021) (34)	40	Serum total bilirubin (mg/dL)	23.4 ± 4.2	15.4 ± 3.4	−34.19
Boonyarittipong et al. (2008) (36)	30	Serum total bilirubin (mg/dL)	14.8 + 1.7	8.4 + 1.7	−43.24
Shimada et al. (2003) (37)	10	Serum total bilirubin (mg/dL)	–	–	–
Kurban et al. (2014) (53)	40	Serum total bilirubin (mg/dL)	21.84 ± 3.6	11.29 ± 1.3	−48.31
Mirnia et al. (2023) (54)	40	Serum total bilirubin (mg/dL)	22.12 ± 2.47	12.52 ± 2.63	−43.40

### Bias analysis

**Figure 2:**
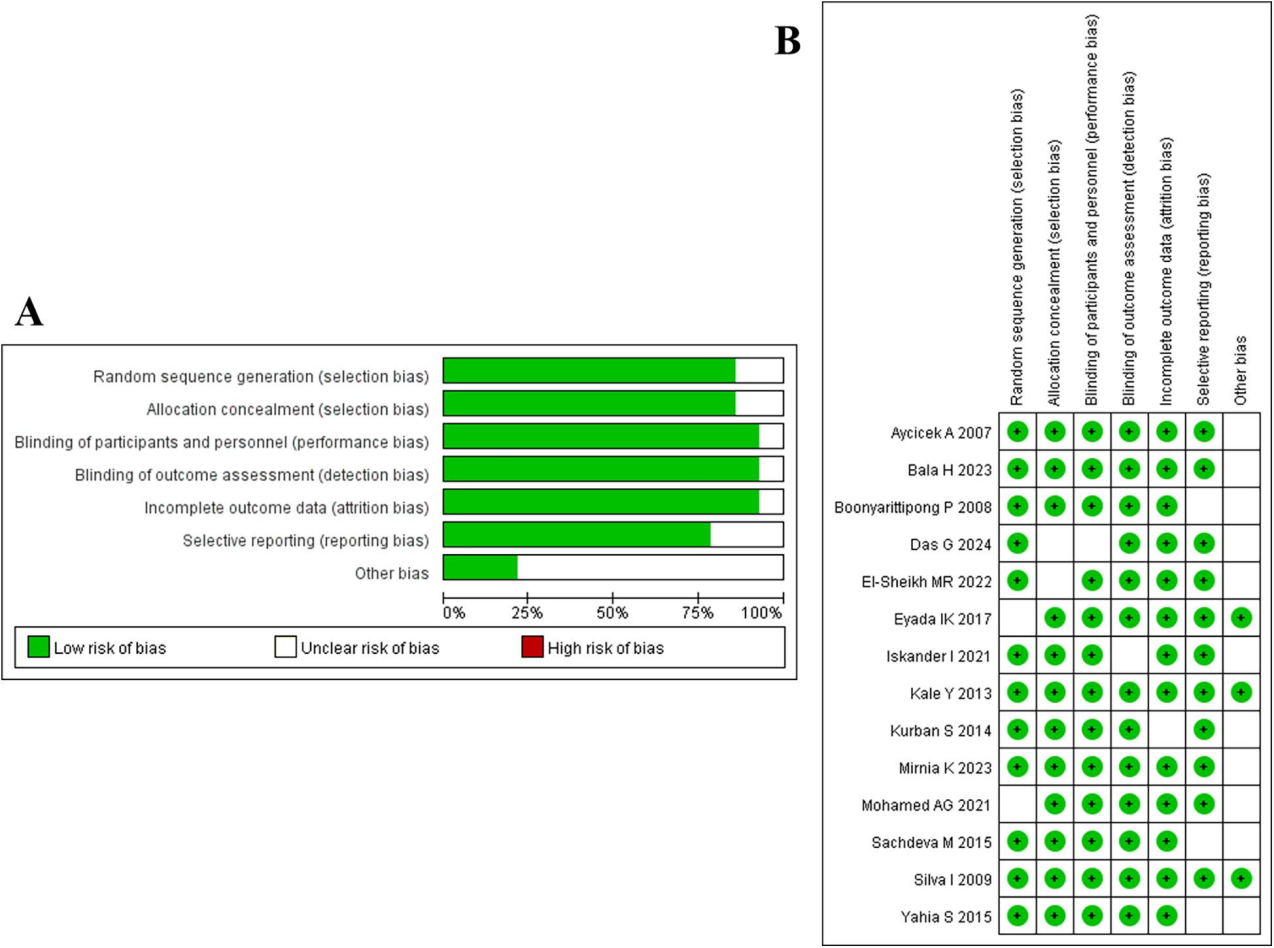
Bias analysis diagram. **(A)** Risk of bias graph. **(B)** Risk of bias summary. Green indicates low risk of bias, red indicates high risk of bias, and blank indicates unclear risk of bias.

The bias analysis results are shown in Figure 2. Random sequence generation (selection bias), allocation concealment (selection bias), blinding of participants and personnel (performance bias), blinding of outcome assessment (detection bias), incomplete outcome data (attrition bias), selective reporting (reporting bias), and other biases were statistically analyzed. Most of the included literature showed low risk of bias across all domains.

### Forest plot

**Figure 3:**
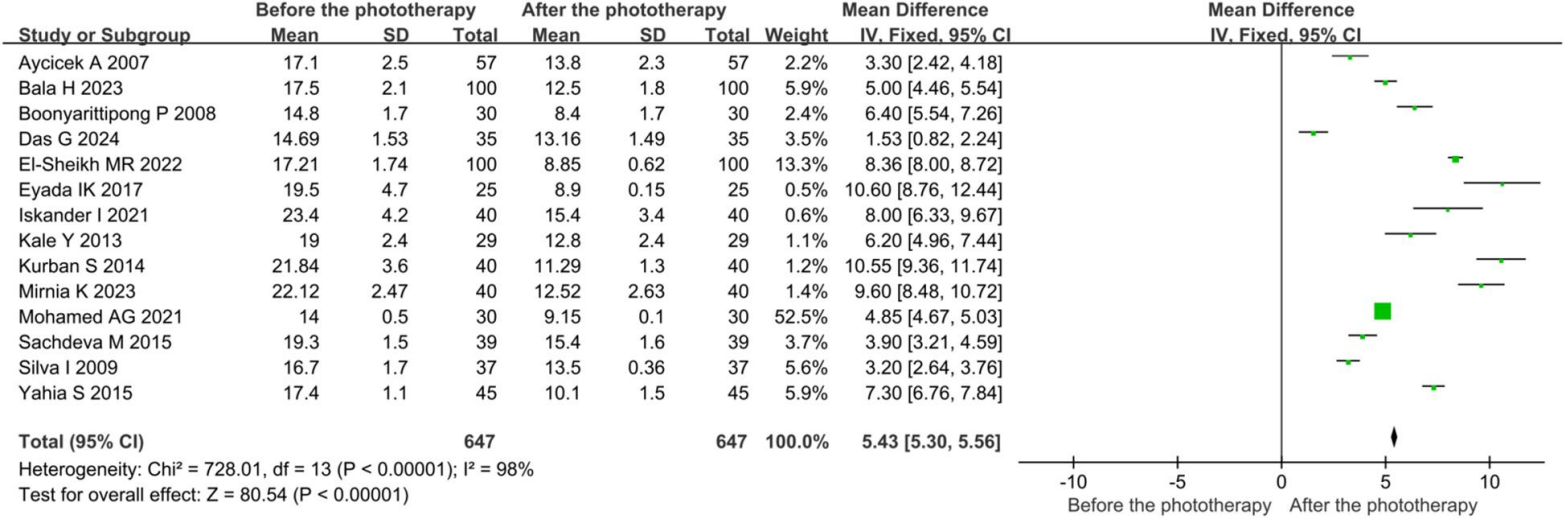
Forest plot. The STB (mg/dL) before and after phototherapy was compared; the left side represents before phototherapy, and the right side represents after phototherapy. The green squares represent the mean difference for each study, and the horizontal lines extending from the squares represent the 95% confidence intervals.

Fourteen included studies with STB (mg/dL) data before and after unidirectional continuous phototherapy were meta-analyzed, and a forest plot was obtained (Figure 3), with the data before phototherapy displayed on the left and the data after phototherapy on the right. We found that the effect values and their confidence intervals of all the included literature were on the right side of the 0 scale line, and the total comparison result was 5.43 (5.30, 5.56). *χ*^2^ = 728.01, df = 13 (*p* < 0.00001), *I*^2^ = 98%, *Z* = 80.54 (*p* < 0.00001) for STB (mg/dL) before and after phototherapy, indicating a statistically significant reduction in bilirubin levels across all studies.

### Funnel plot

**Figure 4:**
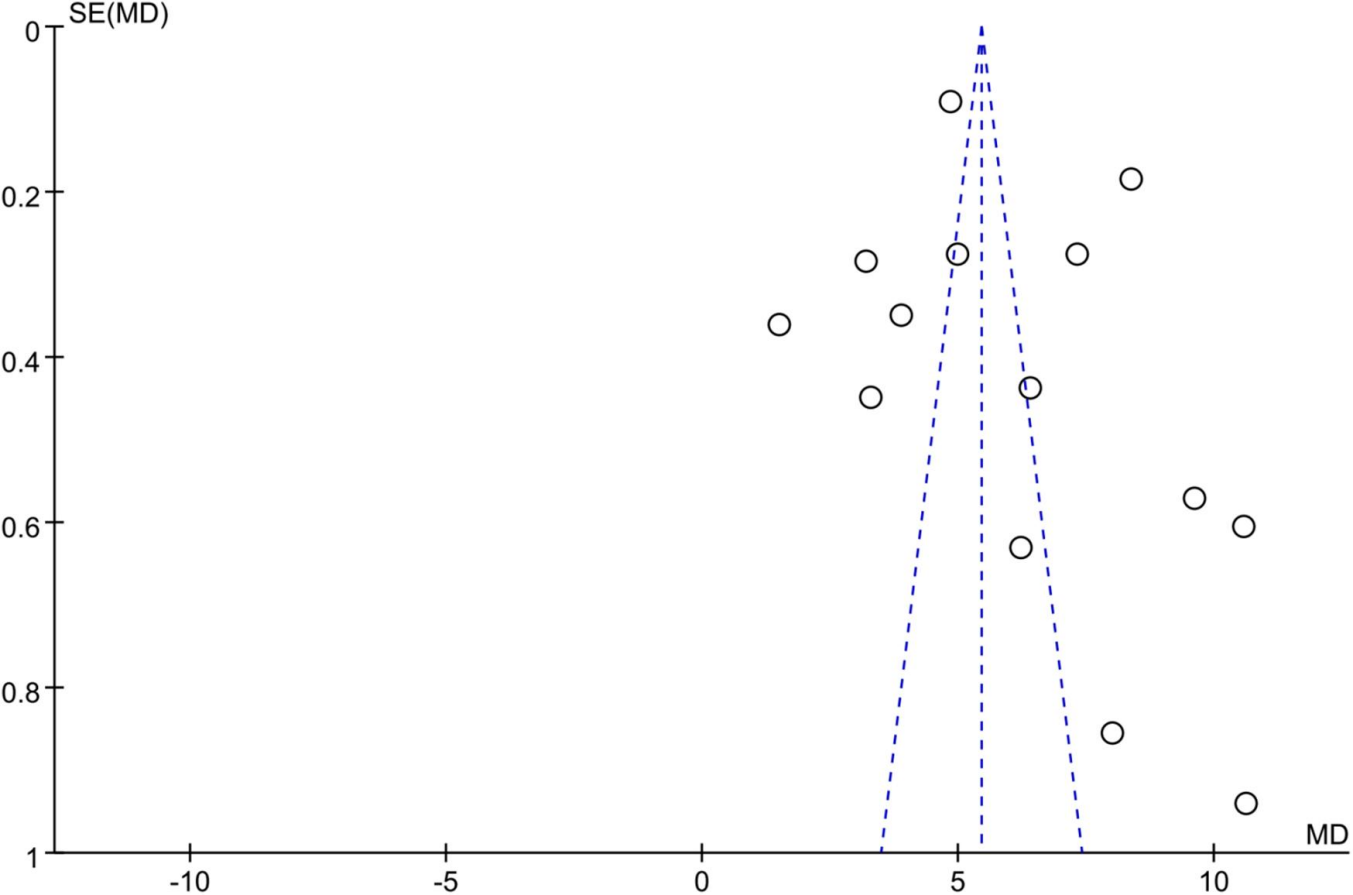
Funnel plot. The vertical line represents the combined effect value with its abscissa >5. The asymmetric distribution of studies indicates potential publication bias.

The funnel plot is shown in Figure 4. The effect size and confidence intervals are completely to the right of the 0 scale line. The horizontal axis represents the mean difference of the effect size, and the vertical axis represents the SE. This study analyzed 647 children with phototherapy. The sample size was large, with a maximum ordinate of 0.8, indicating small variance and small standard error, suggesting reliable results. However, the distribution of studies showed considerable asymmetry in the funnel plot. Most of the studies fell outside the confidence interval, indicating substantial heterogeneity. The asymmetric pattern, with more studies showing larger treatment effects than expected and a relative paucity of studies with smaller or null effects, suggests potential publication bias. This asymmetry may indicate that studies with less favorable results were less likely to be published, a common phenomenon in medical literature. Additionally, the heterogeneity may reflect differences in study quality, phototherapy protocols, device types (LED vs. fluorescent), treatment duration, and patient populations across different countries and continents. Despite these limitations, the overall publication bias risk was assessed as low to moderate, and the consistent direction of effect across all studies supports the efficacy of phototherapy for neonatal jaundice.

### Serum total bilirubin decline rate

**Figure 5:**
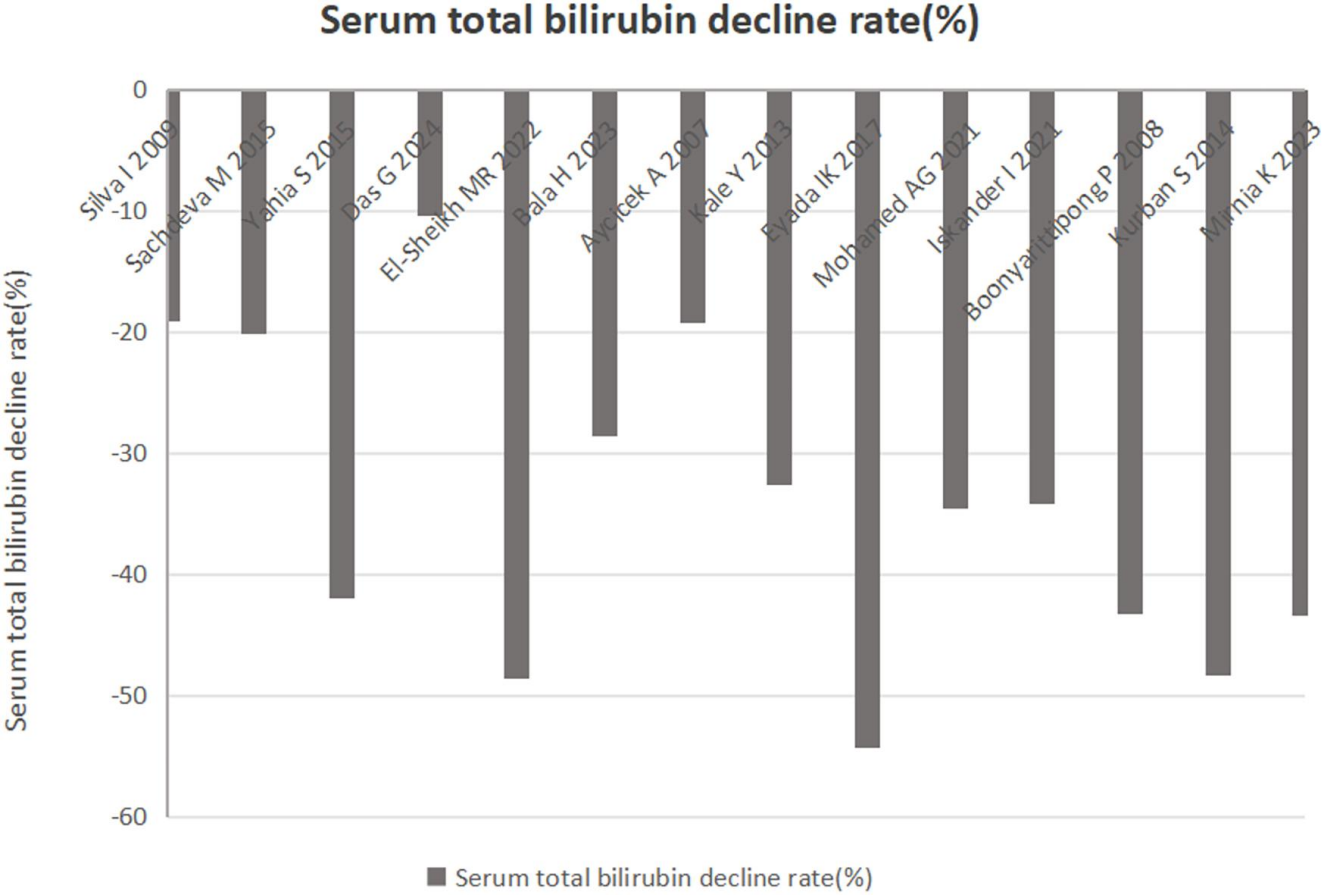
The decrease rate of STB before and after phototherapy. The horizontal axis indicates the literature included in the analysis, and the vertical axis indicates the percentage of STB (mg/dL) decline.

Total serum bilirubin (mg/dL) of full-term neonates before and after phototherapy was documented in 14 included studies, and the decrease rate was calculated and presented in a bar chart for comparison, as shown in Figure 5. All included literature clearly demonstrated that phototherapy resulted in a significant decrease in total bilirubin (mg/dL), with reduction rates exceeding 18% in all studies.

### Sensitivity analysis

**Figure 6:**
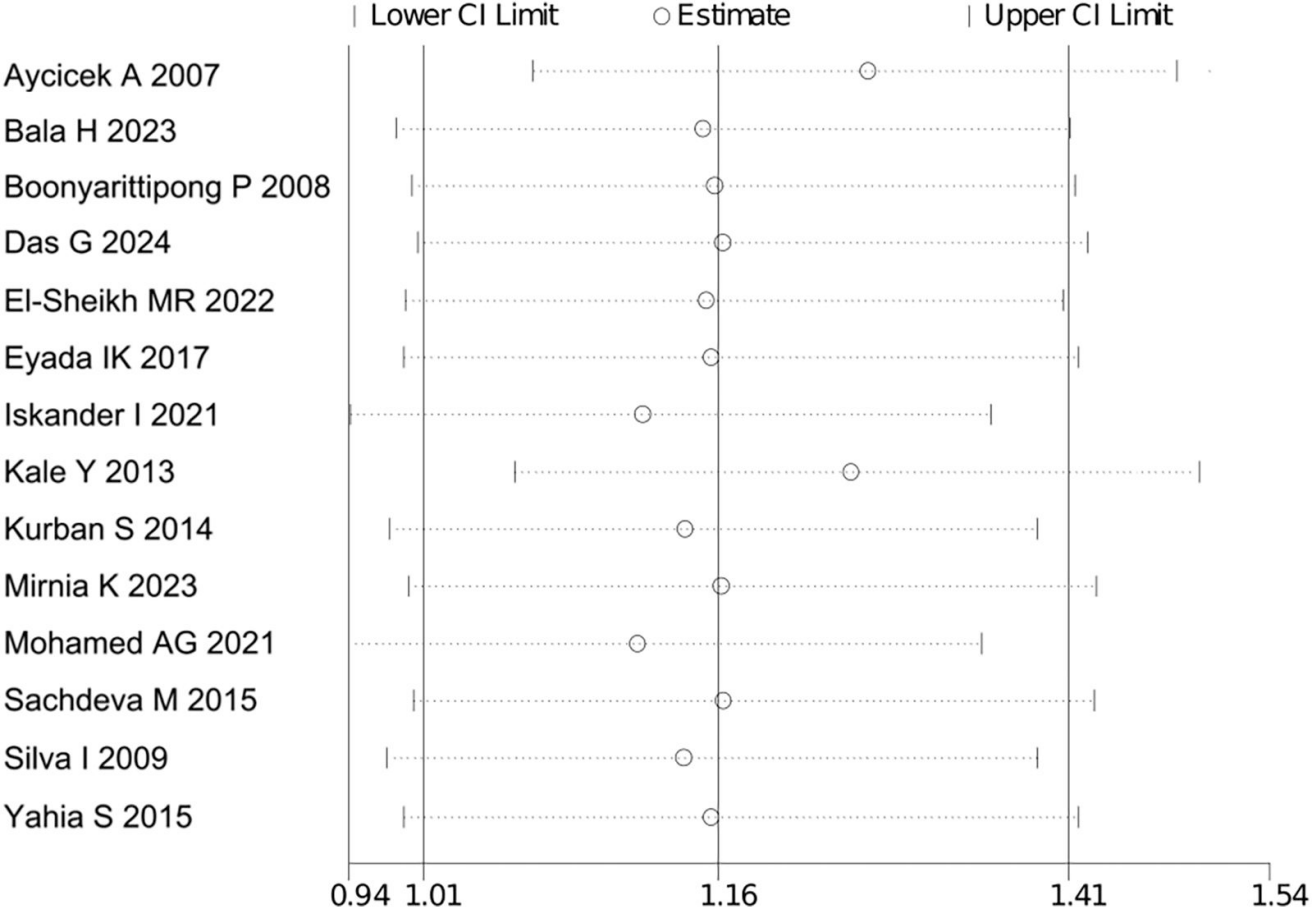
Sensitivity analysis diagram. One-by-one elimination method was used for sensitivity analysis of the included literature to assess the robustness of results and reflect heterogeneity.

The sensitivity analysis is shown in Figure 6. Among the 14 included studies analyzed in the forest plot, the STB (mg/dL) obtained after phototherapy was significantly lower than that before phototherapy, resulting in pre-phototherapy/post-phototherapy values >1. The results of the sensitivity analysis were consistent with the forest plot, and no inversion of results appeared, confirming the robustness of the meta-analysis findings.

### Meta-regression analysis

**Figure 7:**
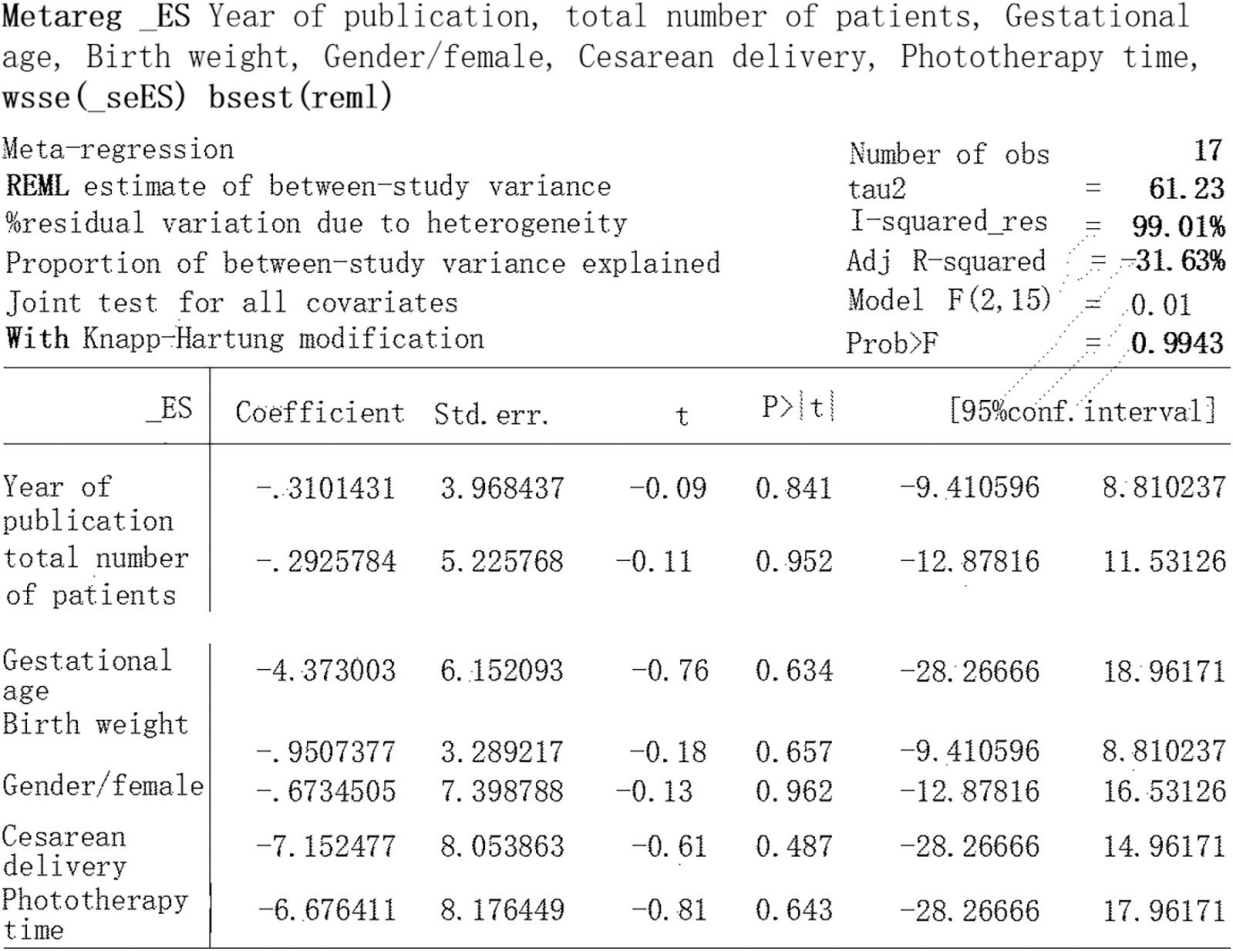
Meta-regression analyses. Year of publication, total number of patients, gestational age, birth weight, gender/female, cesarean delivery, and phototherapy time were included in the meta-regression analysis. All *P*-values were > 0.05, indicating none of these factors significantly contributed to heterogeneity.

Meta-regression analysis is shown in Figure 7. Year of publication, total number of patients, gestational age, birth weight, gender/female, cesarean delivery, and phototherapy time were included in the meta-regression analysis, and all *P*-values were >0.05, indicating that none of these factors were significant sources of heterogeneity in this meta-analysis.

## Discussion

This meta-analysis of 14 studies involving 647 full-term neonates demonstrated that phototherapy significantly reduces STB levels (mg/dL) by 18%–54%, with a mean difference of 5.43 mg/dL (95% CI: 5.30, 5.56). These findings confirm the efficacy of phototherapy as the primary treatment modality for neonatal hyperbilirubinemia (38, 39). The substantial reduction in bilirubin levels across diverse populations and treatment protocols underscores the reliability and effectiveness of this intervention.

According to the AAP Clinical Practice Guideline (21), phototherapy initiation thresholds are based on postnatal age in hours, risk factors (isoimmune hemolytic disease, glucose-6-phosphate dehydrogenase deficiency, asphyxia, lethargy, temperature instability, sepsis, acidosis, or albumin <3.0 g/dL), and total serum bilirubin levels. For example, in well-appearing term neonates at 72 h of age without risk factors, phototherapy is recommended when total serum bilirubin approaches 15 mg/dL, while in neonates with risk factors, the threshold is lower at approximately 13 mg/dL. These evidence-based thresholds guide clinicians in determining appropriate timing for phototherapy initiation to prevent BIND while avoiding unnecessary treatment.

Analysis of phototherapy devices revealed important differences in treatment efficacy. Studies utilizing LED phototherapy devices demonstrated comparable or superior bilirubin reduction rates compared with traditional fluorescent tube systems. LED phototherapy devices offer several advantages, including narrower wavelength emission spectrum (centered at 450–475 nm for optimal bilirubin photoisomerization), higher irradiance levels, longer lamp lifespan, lower heat generation, and reduced energy consumption (40). The modern LED units used in recent studies achieved bilirubin reduction rates of 28%–43% with treatment durations of 48–96 h, while fluorescent tube systems achieved similar reductions but often required longer treatment times (24–72 h) or double-surface phototherapy to reach comparable efficacy. These findings suggest that LED phototherapy devices may offer improved efficiency and patient comfort, though the high heterogeneity in our meta-analysis (*I*^2^ = 98%) indicates that multiple factors beyond device type influence treatment outcomes.

The duration of phototherapy varied considerably across included studies, ranging from 8 to 96 h. Notably, even short-duration phototherapy (8–12 h) in the Iskander et al. (35) and Yahia et al. (32) studies achieved substantial bilirubin reductions of 34% and 42%, respectively. This suggests that intensive phototherapy protocols with high irradiance levels (>30 µW/cm^2^/nm) can achieve rapid therapeutic effects (21). However, most studies employed 24–72 h treatment protocols, which appear to provide a balance between efficacy and practical feasibility. The meta-regression analysis found no significant association between phototherapy duration and treatment effect (*P* > 0.05), likely reflecting the confounding influence of initial bilirubin levels, device types, and irradiance intensity. Future research should focus on optimizing treatment protocols by considering these multiple factors simultaneously.

Regarding adverse effects, our systematic review of included literature found that phototherapy was generally well-tolerated with minimal serious complications. The most commonly reported adverse effects included mild temperature instability (fever or hypothermia), skin rash, and loose stools, all of which resolved upon discontinuation of phototherapy (41, 42). Importantly, recent studies using LED phototherapy devices found no evidence of DNA damage or oxidative stress, addressing earlier concerns about potential genotoxicity. Only three of the 17 included studies specifically investigated adverse effects as a primary outcome. These studies reported no serious adverse events, although minor transient effects such as skin erythema (5%–10% of cases) and increased transepidermal water loss requiring additional fluid supplementation were noted. The paucity of detailed adverse effect reporting in most studies represents a limitation of the current literature. Given the widespread use of phototherapy and the trend toward home-based phototherapy in some healthcare systems, more comprehensive safety monitoring and reporting are warranted.

Geographic and socioeconomic variations in neonatal jaundice management were evident across the included studies. Studies from Middle Eastern countries, El-Sheikh et al. (34), Mosayebi et al. (33), Mirnia et al. (54), showed higher baseline bilirubin levels (mean 17–22 mg/dL) and longer treatment durations compared with studies from East Asian countries, Shimada et al. (37) and Boonyarittipong et al. (36), and Western countries (43). These differences may reflect variations in genetic predisposition to hyperbilirubinemia, differences in clinical protocols for phototherapy initiation, availability of healthcare resources, and cultural practices affecting feeding patterns and early bilirubin screening. In resource-constrained settings, access to phototherapy devices may be limited, requiring prioritization based on clinical severity and implementation of cost-effective alternatives such as filtered sunlight phototherapy in selected cases. The high cesarean delivery rates (30%–83%) reported in several studies may also contribute to delayed breastfeeding initiation and increased jaundice risk. These regional variations highlight the need for context-specific clinical guidelines that consider local epidemiology, healthcare infrastructure, and cultural factors.

For clinical practice, our findings support several recommendations (44). First, phototherapy remains the gold standard treatment for neonatal hyperbilirubinemia, with consistent efficacy across diverse populations and treatment settings. Second, intensive phototherapy using LED phototherapy devices with irradiance ≥30 µW/cm^2^/nm should be considered as first-line treatment for severe hyperbilirubinemia (bilirubin >20 mg/dL), as these protocols achieved bilirubin reductions exceeding 40% within 48–72 h. Third, close monitoring of bilirubin levels is essential during phototherapy, with measurements every 12–24 h to guide treatment duration and detect rebound hyperbilirubinemia. Fourth, attention to hydration status and temperature regulation is crucial, particularly with intensive phototherapy protocols that may increase insensible water loss. Finally, parental education about the safety and necessity of phototherapy can improve treatment adherence and reduce anxiety.

The substantial heterogeneity (*I*^2^ = 98%) observed in our meta-analysis warrants careful interpretation. While meta-regression analysis did not identify specific covariates as sources of heterogeneity, the diversity in phototherapy devices, treatment protocols, patient selection criteria, and outcome measurement timing likely contributes to this variability. The 17-year span of included publications (2003–2024) also encompasses significant technological advances in phototherapy equipment, shifting from fluorescent tubes to LED systems. Additionally, differences in baseline bilirubin levels, timing of phototherapy initiation, and concurrent treatments (such as hydration protocols) may influence outcomes. Despite this heterogeneity, the consistent direction of effect across all studies and the robust sensitivity analysis support the validity of our overall conclusions.

Limitations of this study include the restriction to full-term neonates, excluding premature infants who represent a vulnerable population at high risk for hyperbilirubinemia and bilirubin encephalopathy (kernicterus). We also only analyzed unidirectional continuous phototherapy, excluding studies on intermittent phototherapy, fiber-optic phototherapy, or combination approaches. Three of the 17 included studies lacked complete bilirubin data and could not be included in the meta-analysis. Only peer-reviewed full-text studies were included in this meta-analysis, which may represent a source of potential publication bias, as studies with negative or null results may be less likely to achieve peer-reviewed publication. The asymmetric funnel plot suggests potential publication bias, with possible underrepresentation of studies showing smaller treatment effects. We were unable to perform subgroup analyses by ethnicity, specific jaundice etiology, or detailed device specifications due to insufficient data. Furthermore, long-term developmental outcomes were not assessed in the included studies, and information on the cost-effectiveness of different phototherapy approaches was lacking (45).

Future research should address these gaps through large-scale comparative effectiveness studies of LED vs. fluorescent phototherapy, dose–response studies to optimize irradiance and duration, investigation of combination approaches (such as phototherapy plus pharmacological agents), long-term neurodevelopmental follow-up of treated infants, cost-effectiveness analyses comparing different phototherapy modalities, and studies in premature and low-birth-weight infants (46). Standardized protocols for adverse effect monitoring and reporting would also enhance the evidence base for phototherapy safety. Development of predictive models incorporating genetic, clinical, and treatment factors could enable personalized phototherapy protocols optimized for individual patients.

## Conclusion

Proper phototherapy can significantly reduce the level of STB (mg/dL) and eliminate the symptoms of jaundice in full-term neonates. LED phototherapy devices demonstrate superior or comparable efficacy to traditional fluorescent systems with improved energy efficiency and reduced heat generation. The treatment is safe with minimal adverse effects when properly monitored. The substantial heterogeneity in treatment protocols and outcomes across studies highlights the need for standardized guidelines that consider device type, irradiance intensity, treatment duration, and patient-specific factors. Phototherapy remains the primary treatment for neonatal hyperbilirubinemia and plays a crucial role in preventing bilirubin encephalopathy (kernicterus) and its devastating neurological sequelae.

## Data Availability

The raw data supporting the conclusions of this article will be made available by the authors, without undue reservation.
